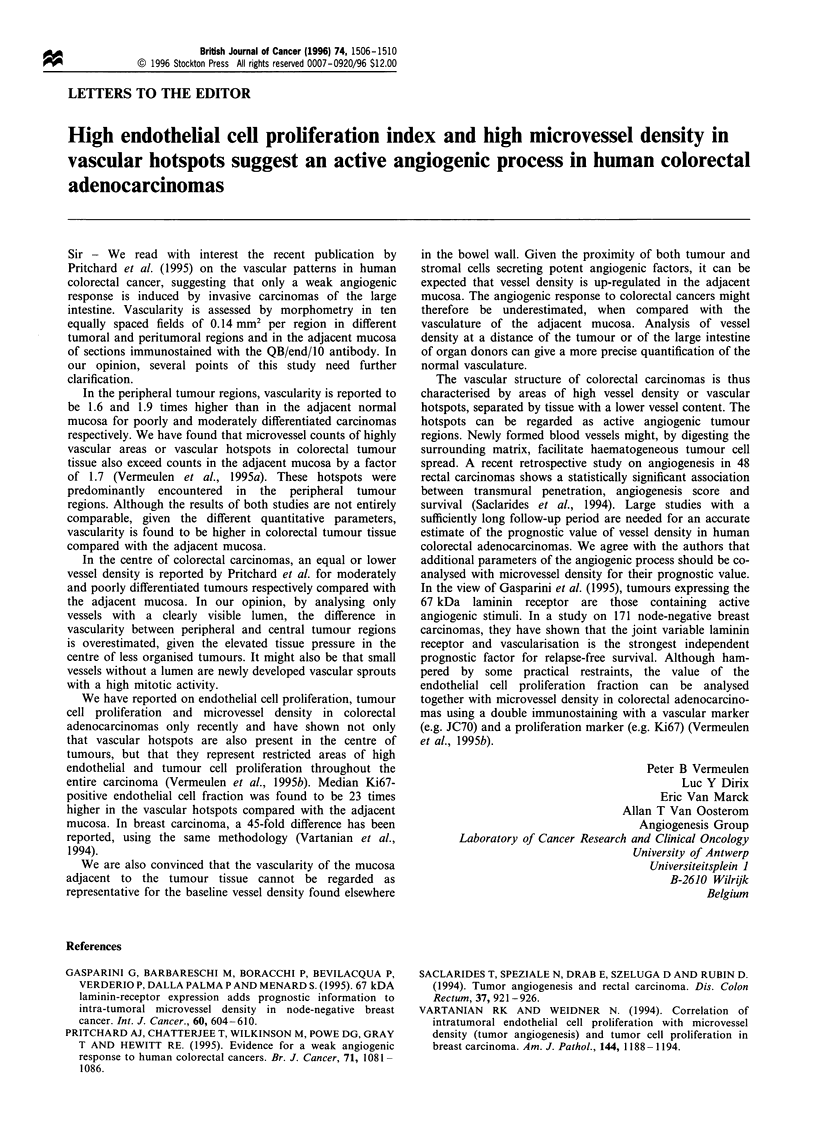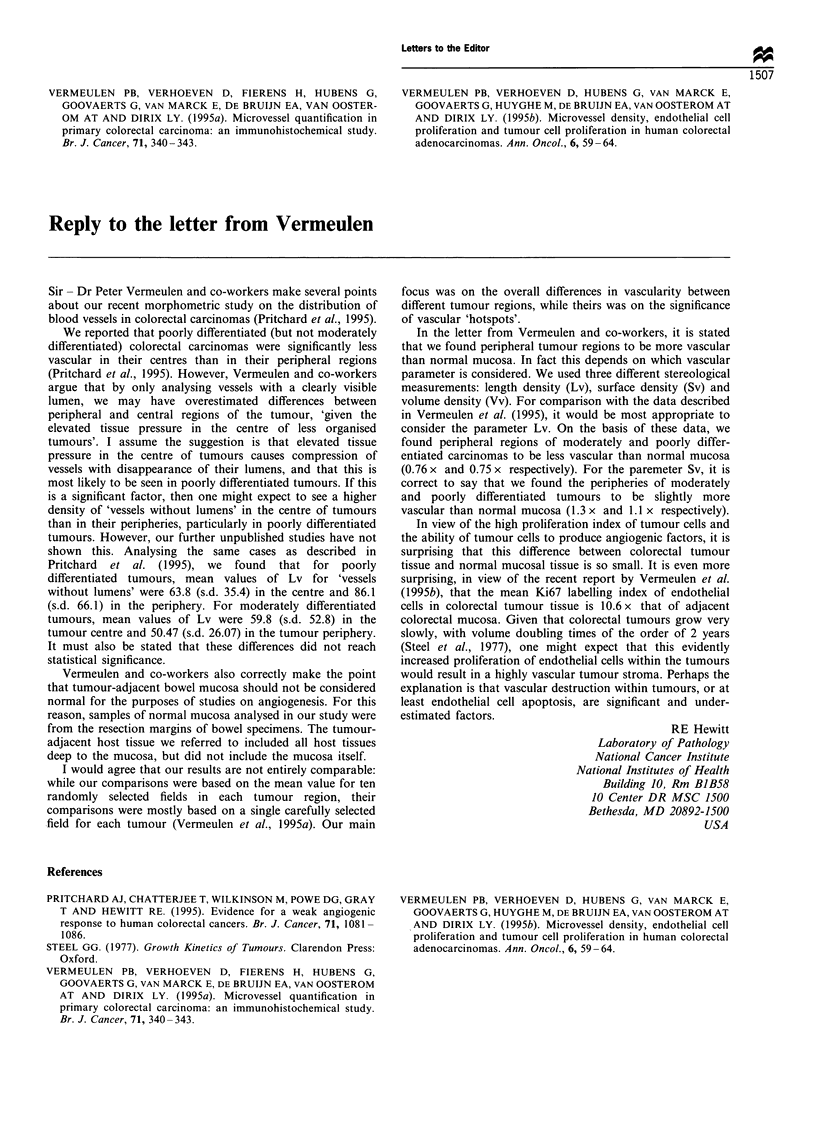# High endothelial cell proliferation index and high microvessel density in vascular hotspots suggest an active angiogenic process in human colorectal adenocarcinomas. Angiogenesis Group.

**DOI:** 10.1038/bjc.1996.574

**Published:** 1996-11

**Authors:** P. B. Vermeulen, L. Y. Dirix, E. Van Marck, A. T. Van Oosterom


British Journal of Cancer (1996) 74, 1506-1510
© 1996 Stockton Press All rights reserved 0007-0920/96 $12.00

LETTERS TO THE EDITOR

### High endothelial cell proliferation index and high microvessel density in vascular hotspots suggest an active angiogenic process in human colorectal adenocarcinomas

Sir – We read with interest the recent publication by Pritchard *et al.* (1995) on the vascular patterns in human colorectal cancer, suggesting that only a weak angiogenic response is induced by invasive carcinomas of the large intestine. Vascularity is assessed by morphometry in ten equally spaced fields of 0.14 $mm^2$ per region in different tumoral and peritumoral regions and in the adjacent mucosa of sections immunostained with the QB/end/10 antibody. In our opinion, several points of this study need further clarification.

In the peripheral tumour regions, vascularity is reported to be 1.6 and 1.9 times higher than in the adjacent normal mucosa for poorly and moderately differentiated carcinomas respectively. We have found that microvessel counts of highly vascular areas or vascular hotspots in colorectal tumour tissue also exceed counts in the adjacent mucosa by a factor of 1.7 (Vermeulen *et al.*, 1995*a*). These hotspots were predominantly encountered in the peripheral tumour regions. Although the results of both studies are not entirely comparable, given the different quantitative parameters, vascularity is found to be higher in colorectal tumour tissue compared with the adjacent mucosa.

In the centre of colorectal carcinomas, an equal or lower vessel density is reported by Pritchard *et al.* for moderately and poorly differentiated tumours respectively compared with the adjacent mucosa. In our opinion, by analysing only vessels with a clearly visible lumen, the difference in vascularity between peripheral and central tumour regions is overestimated, given the elevated tissue pressure in the centre of less organised tumours. It might also be that small vessels without a lumen are newly developed vascular sprouts with a high mitotic activity.

We have reported on endothelial cell proliferation, tumour cell proliferation and microvessel density in colorectal adenocarcinomas only recently and have shown not only that vascular hotspots are also present in the centre of tumours, but that they represent restricted areas of high endothelial and tumour cell proliferation throughout the entire carcinoma (Vermeulen *et al.*, 1995*b*). Median Ki67-positive endothelial cell fraction was found to be 23 times higher in the vascular hotspots compared with the adjacent mucosa. In breast carcinoma, a 45-fold difference has been reported, using the same methodology (Vartanian *et al.*, 1994).

We are also convinced that the vascularity of the mucosa adjacent to the tumour tissue cannot be regarded as representative for the baseline vessel density found elsewhere in the bowel wall. Given the proximity of both tumour and stromal cells secreting potent angiogenic factors, it can be expected that vessel density is up-regulated in the adjacent mucosa. The angiogenic response to colorectal cancers might therefore be underestimated, when compared with the vasculature of the adjacent mucosa. Analysis of vessel density at a distance of the tumour or of the large intestine of organ donors can give a more precise quantification of the normal vasculature.

The vascular structure of colorectal carcinomas is thus characterised by areas of high vessel density or vascular hotspots, separated by tissue with a lower vessel content. The hotspots can be regarded as active angiogenic tumour regions. Newly formed blood vessels might, by digesting the surrounding matrix, facilitate haematogeneous tumour cell spread. A recent retrospective study on angiogenesis in 48 rectal carcinomas shows a statistically significant association between transmural penetration, angiogenesis score and survival (Saclarides *et al.*, 1994). Large studies with a sufficiently long follow-up period are needed for an accurate estimate of the prognostic value of vessel density in human colorectal adenocarcinomas. We agree with the authors that additional parameters of the angiogenic process should be co-analysed with microvessel density for their prognostic value. In the view of Gasparini *et al.* (1995), tumours expressing the 67 kDa laminin receptor are those containing active angiogenic stimuli. In a study on 171 node-negative breast carcinomas, they have shown that the joint variable laminin receptor and vascularisation is the strongest independent prognostic factor for relapse-free survival. Although hampered by some practical restraints, the value of the endothelial cell proliferation fraction can be analysed together with microvessel density in colorectal adenocarcinomas using a double immunostaining with a vascular marker (e.g. JC70) and a proliferation marker (e.g. Ki67) (Vermeulen *et al.*, 1995*b*).

Peter B Vermeulen
Luc Y Dirix
Eric Van Marck
Allan T Van Oosterom
Angiogenesis Group
*Laboratory of Cancer Research and Clinical Oncology*
*University of Antwerp*
*Universiteitsplein 1*
*B-2610 Wilrijk*
*Belgium*

### References

GASPARINI G, BARBARESCHI M, BORACCHI P, BEVILACQUA P, VERDERIO P, DALLA PALMA P AND MENARD S. (1995). 67 kDA laminin-receptor expression adds prognostic information to intra-tumoral microvessel density in node-negative breast cancer. *Int. J. Cancer.*, **60,** 604-610.

PRITCHARD AJ, CHATTERJEE T, WILKINSON M, POWE DG, GRAY T AND HEWITT RE. (1995). Evidence for a weak angiogenic response to human colorectal cancers. *Br. J. Cancer*, **71,** 1081-1086.

SACLARIDES T, SPEZIALE N, DRAB E, SZELUGA D AND RUBIN D. (1994). Tumor angiogenesis and rectal carcinoma. *Dis. Colon Rectum*, **37,** 921-926.

VARTANIAN RK AND WEIDNER N. (1994). Correlation of intratumoral endothelial cell proliferation with microvessel density (tumor angiogenesis) and tumor cell proliferation in breast carcinoma. *Am. J. Pathol.*, **144,** 1188-1194.

VERMEULEN PB, VERHOEVEN D, FIERENS H, HUBENS G, GOOVAERTS G, VAN MARCK E, DE BRUIJN EA, VAN OOSTEROM AT AND DIRIX LY. (1995*a*). Microvessel quantification in primary colorectal carcinoma: an immunohistochemical study. *Br. J. Cancer*, **71**, 340–343.

VERMEULEN PB, VERHOEVEN D, HUBENS G, VAN MARCK E, GOOVAERTS G, HUYGHE M, DE BRUIJN EA, VAN OOSTEROM AT AND DIRIX LY. (1995*b*). Microvessel density, endothelial cell proliferation and tumour cell proliferation in human colorectal adenocarcinomas. *Ann. Oncol.*, **6**, 59–64.